# Identification and Evolution of a Natural Tetr Protein Based on Molecular Docking and Development of a Fluorescence Polari-Zation Assay for Multi-Detection of 10 Tetracyclines in Milk

**DOI:** 10.3390/foods11233850

**Published:** 2022-11-28

**Authors:** Wanqiu Xia, Jing Liu, Jianping Wang

**Affiliations:** College of Veterinary Medicine, Hebei Agricultural University, Baoding 071000, China

**Keywords:** TetR, tetracyclines, recognition mechanism, mutant, fluorescence polarization assay, milk

## Abstract

In this study, the identity of our recently produced natural TetR protein was identified by using the LC-ESI-MS/MS technique, and its recognition mechanisms, including the binding pocket, contact amino acids, intermolecular forces, binding sites, binding energies, and affinities for 10 tetracycline drugs were studied. Then, it was evolved by site-mutagenesis of an amino acid to produce a mutant, and a fluorescence polarization assay was developed to detect the 10 drugs in milk. The sensitivities for the 10 drugs were improved with IC_50_ values decreasing from 30.8–80.1 ng/mL to 15.5–55.2 ng/mL, and the limits of detection were in the range of 0.4–1.5 ng/mL. Furthermore, it was found that the binding affinity for a drug was the critical factor determining its sensitivity, and the binding energy showed little influence. This is the first study reporting the recognition mechanisms of a natural TetR protein for tetracyclines and the development of a fluorescence polarization assay for the detection of tetracyclines residues in food samples.

## 1. Introduction

Due to the broad antibacterial effect, tetracycline drugs (TCs, Table 1) have been used for the treatment of many infectious diseases in human beings since the 1940s [1]. In addition, TCs are also proven to have anti-inflammatory, anti-apoptotic, and anti-malarial effects [2]. However, the wide use of TCs induces many resistant bacterial strains, and the study of their resistant mechanisms becomes an important issue.

In 1974, Levy et al. reported for the first time that the TCs-resistant mechanism was to transfer molecules of TCs out of the cell using a tetracycline repressor (TetR) regulated efflux pump [3]. Thereafter, many experiments were carried out to study this problem. Hillen et al. studied the expression mechanism of the TetR protein at biochemical level [4], and Hinrichs et al. constructed the crystal structure of the TetR(D) of *Acetobacter liquefaciens* and found its two functional domains: a DNA-binding domain and a ligand-binding domain [5]. In the absence of molecules of TCs, the TetR(D) was bound with the DNA-binding domain (TCs-resistance determinant site), and the efflux pump was not expressed. When the molecules of TCs were present, the TetR(D) was bound with TCs and the TetR-TCs complex was dissociated from the DNA-binding domain, so the efflux pump was expressed to transfer the molecules of TCs out of the bacterium [6,7,8,9]. In addition, there were some other papers reporting the TetR’s regulation mechanisms [10,11,12], binding sites [13,14,15] and their potential effects for new drug development [16,17,18], drug metabolism [19], and other functions [20,21].

Still, the most important issue is the binding mechanisms for TCs. In several previous reports, the binding mechanisms of a TetR(D) for a tetracycline [5,6,22] and tetracycline derivative 5a,6-anhydrotetracycline [23,24] were studied, as well as the association/dissociation properties for tetracycline [25,26,27]. From these reports, some important results were obtained. The TetR proteins are dimmers, and each subunit is comprised of ten helices (α1–α10 and α1′–α10′) and two domains: a ligand-binding domain and a DNA binding domain. The binding pocket for the TCs is a tunnel-like cavity, and the main intermolecular force is a hydrogen bond. However, there are still many important questions that have not been answered. For example, can a TetR protein recognize all species of TCs? What is the specific recognition mechanism for each species of TCs? Are the recognition mechanisms the same? To the best of our knowledge, there has been no article reporting these points so far.

It is well known that TCs are also widely used as veterinary drugs in food-producing animals, and their residues in foods of animal origin are potential risks to consumers, e.g., causing gastrointestinal disturbance and allergic reactions. For the protection of consumer health, many countries have set the maximum residue limits for TCs in various food samples, e.g., 100 ng/mL in milk. So, it is necessary to monitor the residues of TCs in foods of animal origin. By now, many analytical methods have been developed to detect their residues. Among the reported methods, an immunoassay is the most commonly used screening tool. In the reported immunoassays for TCs, antibodies are usually used as recognition reagents [28]. However, the procedure of antibody production is tedious, takes a long time, and the cost is high. During the past few years, there have been several papers reporting the use of recombinant TetRs as recognition reagents to develop the microplate method [29,30,31], the test strip [32], and the biosensor [33,34] for the detection of TCs. Results show that these TetR-based methods are comparable to, or better than, the antibody-based immunoassays, and the production of a TetR is simpler, more rapid and costs less than the production of an antibody. So the TetR is a promising recognition reagent for the detection of TCs.

Among different formations of immunoassays, a fluorescence polarization immunoassay is a type of homogeneous method with a simple and rapid assay procedure. For a fluorescence polarization immunoassay, the recognition reagent is directly mixed with the fluorescent tracer and the analyte solution to be incubated for several minutes, and then the fluorescence polarization value is excited to quantify the analyte. In other words, the coating, blocking, and washing steps that are required in the conventional immunoassay are avoided, so the assay can be finished within several minutes. Therefore, this method has been used to determine different analytes [35]. To the best of our knowledge, however, there has been no article reporting fluorescence polarization immunoassay for the detection of TCs so far.

In our recent study, a type of natural TetR protein capable of recognizing 10 TCs was obtained [36], but its identity was not identified and its recognition mechanisms for TCs are unknown. In the present study, its identity was characterized by using the LC-ESI-MS/MS technique, and its recognition mechanisms and affinities for the 10 TCs shown in Table 1 were thoroughly studied by using molecular docking and surface plasmon resonance, respectively. To further improve its recognition ability for TCs, a key contact amino acid was changed by using a site-directed mutagenesis technique. Then, the obtained mutant was used to develop a fluorescence polarization assay (FPA) for the detection of the 10 TCs in milk.

## 2. Materials and Methods

### 2.1. Reagents

The standards of tetracycline (TC), chlortetracycline (CTC), minocycline (MC), doxycycline (DC), oxytetracycline (OTC), demeclocycline (DMC), and tigecycline (TIC) were from Sigma-Aldrich (St. Louis, USA). Methacycline (MTC) was from J&K Scientific Ltd. (Beijing, China). Lymecycline (LMC) was from Toronto Research Chemicals (Toronto, Canada). Sancycline (SC), fluoresceinamine (FA), and fluorescein isothiocyanate (FITC) were purchased from Shanghai Yuanye Biological Technology Co., Ltd. (Shanghai, China). All the biological reagents (isopropyl-beta-D-thiogalactopyranoside, culture medium, express vector *pET32a*) and the kits (EasyPure Quick Gel Extraction Kit, Fast MultiSite Mutagenesis System, sodium dodecyl sulfate polyacrylamide gel electrophoresis (SDS-PAGE) gel) were the same as our recent report [29].

### 2.2. Identification of Natural TetR Protein

The identity of our recently obtained natural TetR was identified by using the LC-ESI-MS/MS technique, and the procedures were according to our recent report [37]. After this experiment, its gene sequence and amino acid sequence were obtained from NCBI.

### 2.3. Molecular Docking

The amino acid sequence of the natural TetR was retrieved in PDB, and its 100% homological model (PDB ID: 4AC0) was used to dock with the 10 TCs, respectively, by using YASARA 16.2.18 (YASARA Biosciences GmbH, Austria). During the molecular docking, the binding pocket was located, and several important parameters including key contact amino acids, main intermolecular forces, total binding energies, and specific binding sites for each drug were determined.

### 2.4. Surface Plasmon Resonance (SPR)

For confirmation of the molecular docking results, the affinities of the natural TetR for the 10 TCs were tested, respectively, by using the Berthold bScreen LB 991 Microarray System, and the procedures were according to our recent report [38]. After the experiments, the association constant (Ka), the dissociation constant (Kd), and the equilibrium dissociation constant (KD = Kd/Ka) for each drug were calculated. For convenience in comparing their affinities, the absolute affinity constant (KA = Log2(KD)) for each drug was also calculated.

### 2.5. Mutation of Natural TetR

Based on the results of molecular docking, the amino acids in the binding pocket were subjected to saturate virtual mutation by using YASARA. The mutagenesis site was selected according to the virtual mutation for demeclocycline (DMC), which was because the natural TetR showed the highest binding energy to it. In this study, if an amino acid was substituted with another amino acid, and the obtained virtual model showed improved binding for DMC, then this amino acid was selected as the mutagenesis site. During the virtual mutation, HIS139 was found to be the optimal mutagenesis site, and it was substituted with THR to produce the mutant.

The primers were designed according to the virtual model: mutant _for_, TAATACGACTCACTATAGG; mutant _rev_, GCTAGTTATTGCTCAGCGG. The amplified gene was inserted into *pET28a* to construct the express vector that was transformed into *E. coli* Rosetta-gami(BL21) to express the mutant, and these procedures were according to our recent report [30]. The obtained TetR mutant was characterized by using SDS-PAGE and western blotting according to our recent report [36], and its recognition ability for the 10 TCs was evaluated by the following FPA method. Furthermore, its recognition mechanisms and affinities for the 10 TCs were also determined as described above.

### 2.6. Synthesis of Fluorescent Tracers

**Figure 1:**
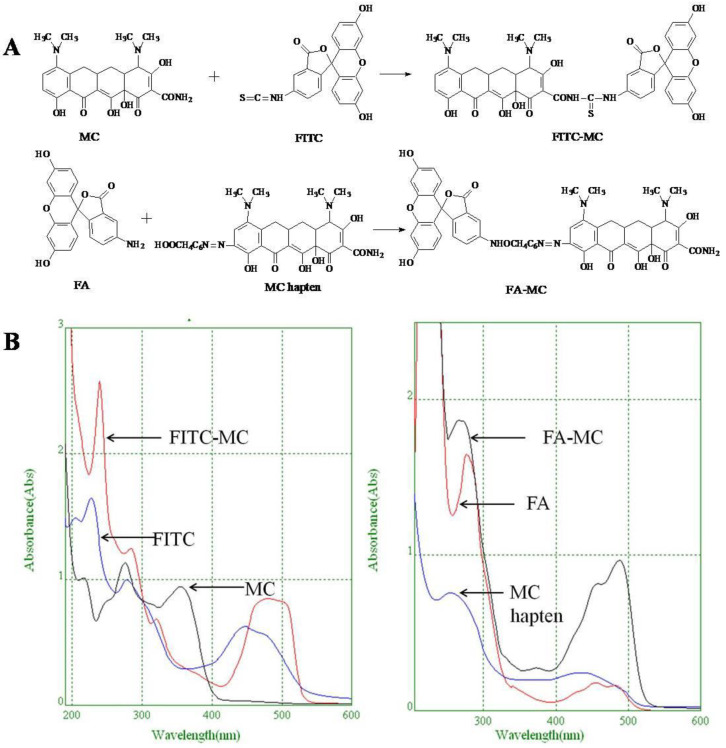
(**A**) Synthetic routs and (**B**) UV scanning diagrams of FITC-MC and FA-MC.

The first fluorescent tracer FITC-MC (Figure 1A) was synthesized in our recent report by coupling minocycline (MC) with fluorescein isothiocyanate (FITC) [29]. The second fluorescent tracer FA-MC (Figure 1A) was synthesized by coupling the MC hapten (obtained in our recent report [36]) with fluoresceinamine (FA) by using the active ester method. Briefly, the MC hapten (5 mg), N-ethyl-N’-(3-dimethylaminopropyl) carbodiimide hydrochloride (4.1 mg) and N-hydroxysuccinimide (2.4 mg) were added into 2 mL DMF to be stirred for 4 h. This solution was added into 2 mL dimethylformamide containing 4 mg FA to be stirred overnight. The obtained mixture was separated on a homemade thin layer chromatography plate, and the target lane was scraped and eluted with methanol. The obtained solution containing FA-MC was stored at 4 °C before use.

### 2.7. Development of FPA Method

The FPA method was performed as follows. A total of 50 μL mutant, 50 μL TCs solution, and 50 μL fluorescent tracer were added into the wells of a 96-well microplate (in triplicate) and incubated for 2 min. Then the fluorescence polarization (*FP*) value of each well was recorded (λ_ex_ 485 nm, λ_em_ 528 nm, emission cutoff of 515 nm). During the experiments, the two fluorescent tracers were compared, and several other parameters were optimized, including tracer concentration, mutant concentration, Mg^2+^ concentration and incubation time. The 10 TCs and several other classes of drugs were all tested by this method. The competitive curve was developed by plotting the *FP/FP_0_* values (the fluorescence polarization value at each concentration divided by that of zero concentration) versus the concentrations of the TCs (log C). Based on the developed competitive curve, the half of inhibition (IC_50_) and the limit of detection (IC_10_) for each drug were determined, respectively.

### 2.8. Method Application

For evaluation of the method, some blank milk samples were collected from several controlled dairies known to be free from TCs, and the 10 TCs were fortified into the blank samples to be extracted as in our recent report [36] and determined by the present FPA method, respectively. Finally, 60 real milk samples purchased from several local supermarkets were analyzed as described above and confirmed by our recently reported ultra-performance liquid chromatography method (UPLC) [39].

## 3. Results and Discussions

### 3.1. Characterization of TetR Identity

In our recent report, the natural TetR was produced by using a photoaffinity labeled activity-based protein profiling probe [36], but its specific identity was unknown. It is well known that LC-ESI-MS/MS is the commonly used technique to identify a newly found protein. In the present study, the identity of the natural TetR was identified by using this technique. The results showed that nine unique peptides of the TetR family were obtained, and their amino acid sequences, molecular weights, and mass spectrometry results were shown in Table S1 and Figure S1 (Supplementary Material). After retrieving the Uniprot Homo Database, it was found that only a TetR(B) protein (NCBI ID: WP_000088605.1) simultaneously contained the nine peptide fragments. This TetR contained 207 amino acids, and the molecular weight was 23.354 kDa.

As shown in Figure S2, its amino acid sequence was same as the *Escherichia coli*. TetR (PDB ID: 4AC0). Compared with the TetR proteins from other bacterial strains, the 100% conserved amino acids were the same, but the amino acids at other positions were different. Based on these results, it could be said that the natural TetR protein produced in our recent report was completely consistent with the previously produced TetR protein. So, the photoaffinity labeled activity-based protein profiling probe could also be used to produce the natural receptor of other drugs.

### 3.2. Recognition Mechanism of Natural TetR

**Figure 2:**
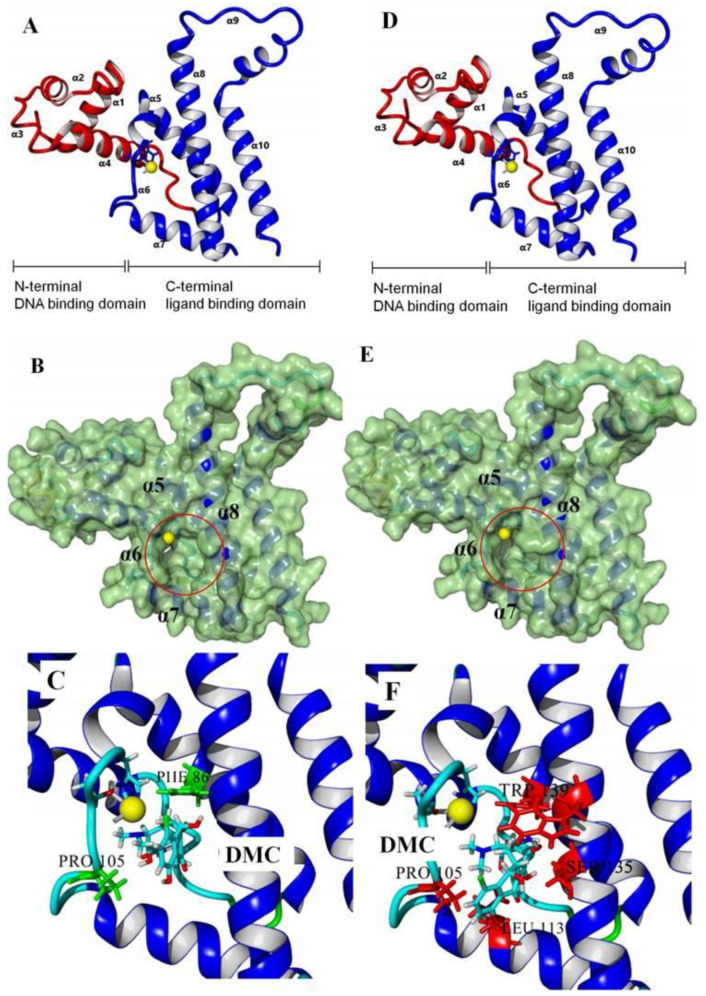
(**A**) The 3D structure of the natural TetR, (**B**) its binding pocket, and (**C**) its docking complex with DMC. (**D**) The 3D structure of the mutant, (**E**) its binding pocket, and (**F**) its docking complex with DMC.

In this study, the 100% homological model of the natural TetR (PDB ID: 4AC0) was used for molecular docking. As shown in Figure 2A, the natural TetR (monomer) contained 10 α-helices, and it could be divided into a DNA binding domain (α1 to α4) and a ligand binding domain (α5 to α10), which was the same as the previous reports [5,6,7,8,9,22,23,24]. As shown in Figure 2B, the binding pocket was a tunnel-like cavity that was constructed by α5, α6, α7, and α8 helices. Then the 10 TCs were docked with the model, respectively. The docking results are shown in Table 1, and the docking complexes are shown in Figure 2C and Figure S3 (Supplementary Material).

As shown in Figure 2C and Figure S3, the 10 TCs could all be docked into the pocket, and the D-rings of their molecules were deeply in the pocket. As shown in Table 1, PHE86 (α5), PRO105 (α6), LEU113 (α7), and LEU131 (α8) were the main contact amino acids. PRO105 was involved in the bindings with all of the 10 TCs, PHE86 and LEU113 were involved in the bindings with 8 TCs, and LEU131 was involved in the bindings with 7 TCs. During the dockings, it was found that hydrophobic interaction was the main intermolecular force that was involved in the bindings with all of the 10 TCs, and the binding sites were mainly on their D rings (Table 1). Furthermore, a hydrogen bond was involved in the bindings with five TCs (Table 1), so it was the secondary force. Due to these similar interactions, the binding energies for the 10 TCs were in a narrow range (7.58–8.56 kcal/mol), i.e., the TetR showed comparable bindings for the 10 drugs.

By now, there have been several reports studying the association/dissociation properties of the TetR protein for tetracycline [25,26,27]. In the present study, the Kd, Ka, and KD values for the 10 TCs were calculated, respectively (Table S2), and for convenience the absolute affinity constants (KA) were used to evaluate the TetR-TCs affinities. In a previous article, the authors reported that Mg^2+^ showed some influences on TetR-tetracycline binding [27], so the affinities when adding and without adding Mg^2+^ were all calculated in the present study. As shown in Table S2, the KA values for the 10 TCs when adding Mg^2+^ were in the range of 17.7–25.1, and the values without adding Mg^2+^ were in the range of 17.0–26.5. This meant that the presence or absence of Mg^2+^ showed little influence on the TetR-TCs bindings. From these KA values, it could also be said that the TetR showed comparable bindings for the 10 drugs.

By now, there have been several reports studying the TetR-TCs intermolecular mechanism (TetR(D) for tetracycline) [5,6,22]. In those reports, the authors showed that a hydrogen bond was the main intermolecular force, the main binding site was at the D ring of tetracycline, and Mg^2+^ was required. The present paper studied the recognition mechanisms of a natural TetR for 10 TCs for the first time, and the results were more comprehensive than the previous studies.

### 3.3. Characterization of TetR Mutant

For obtaining a TetR mutant with improved recognition ability for TCs, HIS139 was mutated to TRP139 based on the virtual mutation for DMC. As shown in Figure 2D, the virtual model also contained 10 helices, and its 3D structure was almost the same as the natural TetR. Its binding energy for DMC showed little change (Table 1), but its binding pocket narrowed (Figure 2E), the number of contact amino acids increased (SER135 (α8) and TRP139 (α8), Figure 2F), and the number of binding sites in the DMC molecule increased (Table 1). These results suggested that the mutant-DMC affinity increased. Then the virtual model was docked with the other nine TCs, respectively, and the results were shown in Table 1 and Figure S3.

As shown in Table 1, hydrophobic interaction was still the main intermolecular force that was involved in the bindings with all of the nine TCs, and a hydrogen bond was only involved in the bindings with four TCs. Similar to the mutant-DMC binding, the numbers of contact amino acids for eight TCs (except TC) increased, the two new contact amino acids (SER135 and TRP139) involved in the bindings with seven TCs, and the binding sites in the eight TCs all increased, though the binding energies for the nine drugs changed a little (Table 1). As shown in Figure S3, the nine TCs could all be docked into the mutant pocket, the positions of seven TCs (except MTC and LMC) in the pocket were deeper than their positions in the natural TetR pocket, and the mutant-TCs bindings were tighter than the natural TetR-TCs bindings. So, the virtual model was expressed for further evaluation.

**Figure 3:**
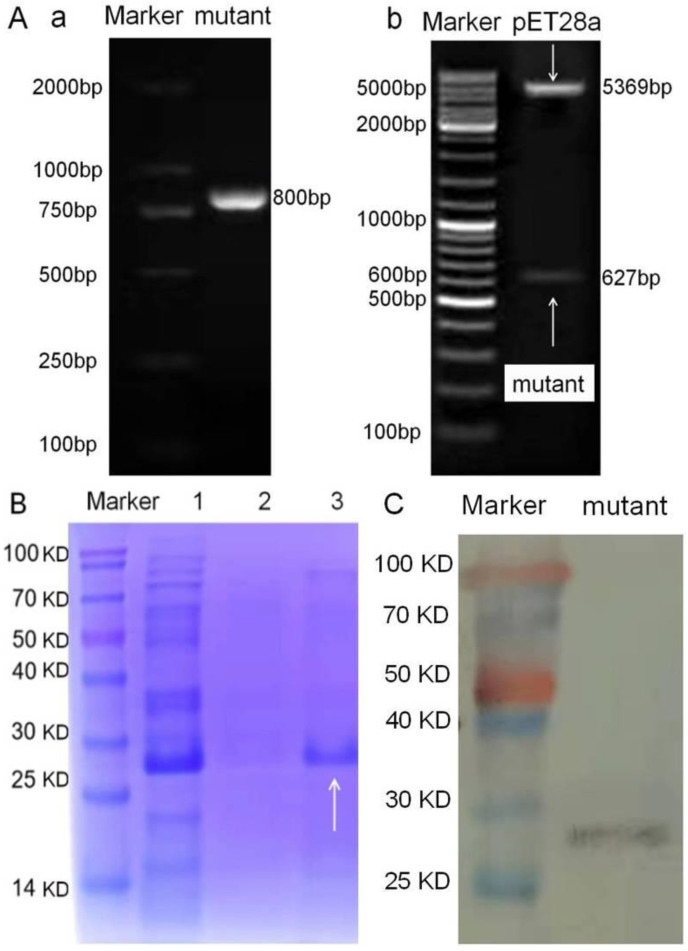
(**A**) Agarose gel electrophoresis results of (**a**) The TetR mutant (containing 6 × His-tag, about 170 bp) and (**b**) express vector pET28a-TetR mutant. (**B**) SDS-PAGE result of the TetR mutant (lane 1, bacterial whole protein; lane 2, purified inclusion body; lane 3, purified supernatant). (**C**) Western blotting result of the TetR mutant.

As shown in Figure 3(Aa), the agarose gel electrophoresis analysis showed that the expected mutant gene was obtained (about 800 bp, containing 6 × His-tag 170 bp). As shown in Figure 3(Ab), the expected genes of pET28a (5369 bp) and the mutant (627 bp) were obtained after restriction digestion with *BamHI* and *XhoI*, indicating the express vector was obtained. As shown in Figure 3B, the SDS-PAGE analysis showed that its molecular weight was about 27.354 kDa (containing 6 × His-tag, about 4 kDa), consistent with the LC-ESI-MS/MS result. As shown in Figure 3C, the western blotting result based on the anti-TetR antibody showed that a TetR protein was obtained. These results proved that the TetR mutant was obtained.

Furthermore, its affinity parameters for the 10 TCs were also calculated. As shown in Table S2, the KA values for the 10 TCs when adding Mg^2+^ (23.9–33.3) were generally similar to those where Mg^2+^ (20.3–33.1) was not added, indicating the presence or absence of Mg^2+^ also showed little influence on its binding for TCs. As shown in Table 1, the KA value for each drug (adding Mg^2+^) when using the mutant was higher than that when using the natural TetR, indicating the natural TetR was evolved successfully and the mutant showed improved affinities for the 10 TCs.

### 3.4. Characterization of Fluorescent Tracers

FITC and FA are the commonly used fluorescent agents, so they were used to synthesize the two fluorescent tracers in the present study (Figure 1A). As shown in Figure 1B, the UV scanning diagrams showed that both FITC-MC and FA-MC contained the characteristic peaks of fluorescein and MC, indicating the two tracers were obtained.

### 3.5. Comparison of the two Fluorescent Tracers

**Figure 4:**
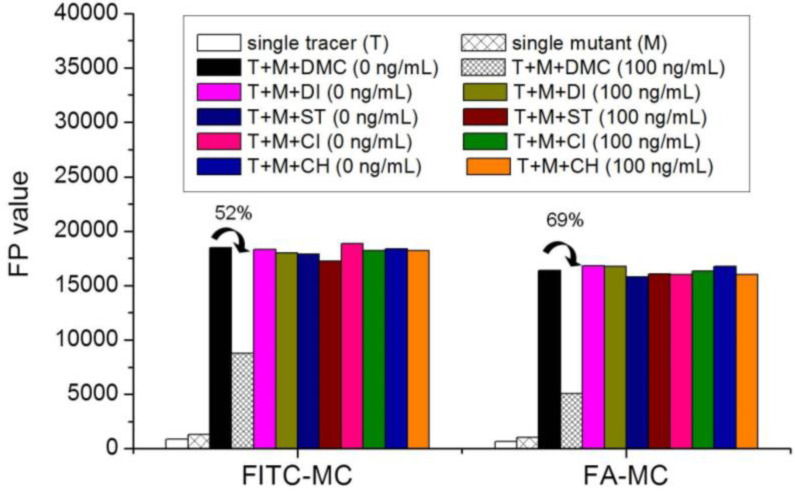
*FP* values when testing DMC and other drugs by using the two fluorescent tracers (DI = diazepam, ST = streptomycin, CI = ciprofloxacin, CH = chlorpromazine; *n* = 5).

In order to verify whether the two fluorescent tracers and the mutant could be used to develop a FPA method, DMC and the mutant were mixed with FITC-MC and FA-MC to perform the assay, respectively. As shown in Figure 4, when DMC was 0 ng/mL the *FP* values of a single tracer and a single mutant were all negligible, but the *FP* values of their mixtures were all high, indicating the two tracers could bind with the mutant to develop an FPA method. However, the *FP* value when using FITC-MC was higher than that when using FA-MC. The molecular docking showed that the binding sites in the MC molecule were around its D ring (Table 1). As shown in Figure 1A, the two fluorescent tracers were actually synthesized by coupling fluorescein with MC at two opposite positions. In FITC-MC, the D ring of MC was free, whereas the D ring of MC in FA-MC was linked with fluorescein, so FITC-MC showed higher binding ability for the mutant than FA-MC, thus obtaining the higher *FP* value. These results proved that the results of molecular docking were correct.

As shown in Figure 4, when the DMC concentration was 100 ng/mL, the *FP* values when using the two tracers all decreased, but the inhibition ratio (1-*FP/FP_0_*) when using FA-MC (69%) was higher than when using FITC-MC (52%). This was because FA-MC showed weaker binding for the mutant than FITC-MC, thus improving the DMC’s binding for the mutant to obtain the high inhibition ratio. This indicated that the use of FA-MC could achieve a higher sensitivity, so it was used for the subsequent experiments. During the experiments, several other classes of drugs (diazepam, streptomycin, ciprofloxacin, and chlorpromazine) were also tested by this method. As shown in Figure 4, the *FP* values when their concentrations were 0 and 100 ng/mL were comparably high no matter which tracer was used, indicating the mutant was only specific for TCs.

### 3.6. Optimization of FPA

For obtaining the best method performance, several parameters were optimized with DMC as the representative. Firstly, the concentrations of FA-MC and the mutant were optimized. During the experiments, the FA-MC and the mutant at different concentrations were mixed with the same concentration of DMC to perform the assay. As shown in Figure S4A, the inhibition ratio of DMC was the highest when using 1:500 of FA-MC and 1:2000 of the mutant, so the two parameters were selected as the optimal conditions. Secondly, the concentration of Mg^2+^ (MgCl_2_) was optimized. As shown in Figure S4B, the inhibition ratio of DMC was the highest when using 8 mM MgCl_2_, so this parameter was selected as the optimal condition. Thirdly, the competition time was optimized. As shown in Figure S4C, the inhibition ratio of DMC reached a plateau when the competition time increased to 2 min, so this parameter was selected as the optimal condition.

### 3.7. Method Performances

**Figure 5:**
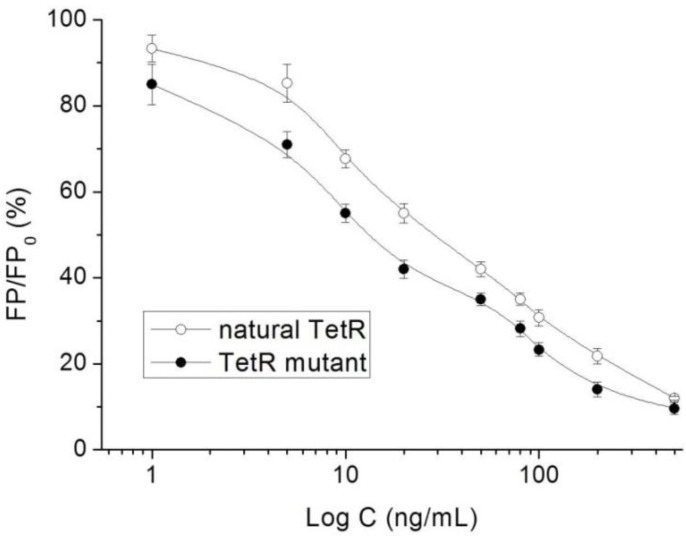
Competitive curves of DMC when using the natural TetR and the mutant (*n* = 5).

This is the first study reporting an FPA method for the determination of TCs. Under the optimal conditions, the 10 TCs were diluted with the extracts of a blank milk sample to be analyzed, respectively. As shown in Table 1, the IC_50_ values for the 10 TCs were in the range of 15.5–55.2 ng/mL, and the limits of detection were in the range of 0.4–1.5 ng/mL. During the experiments, the natural TetR was also used to perform the FPA method as described above. As shown in Table 1, the IC_50_ values for the 10 TCs when using the natural TetR were in the range of 30.8–80.1 ng/mL, indicating the natural TetR was evolved successfully and the mutant showed improved sensitivities for the 10 TCs. The representative competitive curves of DMC when using the natural TetR and the mutant are shown in Figure 5.

**Figure 6:**
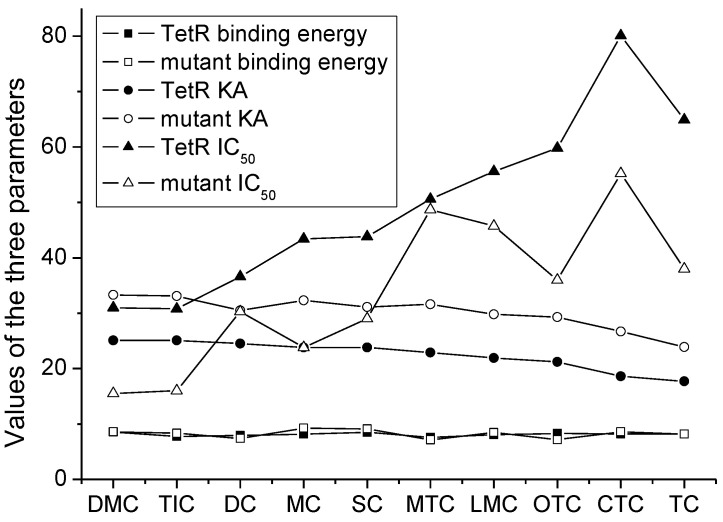
Change trends of the binding energy, absolute affinity constant (KA), and IC_50_ for the 10 TCs when using the natural TetR and the mutant.

For a comprehensive comparison of the two proteins, the binding energy, KA, and IC_50_ of the 10 TCs when using the natural TetR and the mutant were integrated. As shown in Figure 6, the binding energies of the 10 drugs were comparable no matter which protein was used, indicating the binding energy showed little influence on their recognition of different species of TCs. However, the KA values of the 10 drugs when using the mutant were all higher than when using the natural TetR, and the IC_50_ values of the 10 drugs when using the mutant were all lower than when using the natural TetR. These results showed that the natural TetR was evolved successfully, and the affinity for a drug determined the sensitivity for it.

### 3.8. Method Application

The 10 TCs were fortified into the blank milk samples to be analyzed, respectively. As shown in Table 2, their recoveries ranged from 71.5% to 95.4%, and the coefficients of variation ranged from 5.1% to 16.3%. Finally, the 60 real milk samples were analyzed by the present FPA method. Results showed that only two real samples were determined as positive (35 and 61 ng/mL, expressed as DMC), but the specific drug species could not be identified. The results from the UPLC method showed that the two samples contained residues of TCs (TC 27 ng/mL, OTC 55 ng/mL), and other real samples were all confirmed as negative samples. Therefore, the positive results of the present FPA method should be confirmed with an instrumental method to identify the specific drug due to the mutant’s broad recognition ability. Still, this FPA method could be used for rapid screening of the residues of the 10 TCs in a large number of milk samples.

### 3.9. Comparison with Related Methods

For the first time, this study developed a TetR mutant-based FPA method for the detection of TCs. For comparison with related methods, the previously reported TetR-based analytical methods for TCs were listed in Table 3. Firstly, the microplate method [31], dipstick-based method [32] and chemiluminescence methods [29,36] required coating, sample-loading, several cycles of incubating steps and a substrate system, which were tedious. Secondly, the fluoroimmunoassay also required a coating step, which was inconvenient [30]. Thirdly, the electrochemical chip [33] and the flow-based chemiluminescence method [34] required a sophisticated preparation process. Fourthly, the detection spectrum of the present method was broader than those methods except in our recent report [36]. Fifthly, the assay time of the present method was shorter than all of those methods. Therefore, the present FPA method showed better performances than those related methods. With the guidance of the present study, more research about the evolution of the receptors of other classes of drugs by using similar or more advanced techniques should be reported in the future, and other novel analytical methods based on receptor could also be reported.

## 4. Conclusions

In this study, the identity of a natural TetR protein was characterized, and its recognition mechanisms for 10 TCs were studied. It was found that the TetR showed similar binding sites, contact amino acids, and intermolecular forces for the 10 drugs. Then, the natural TetR was evolved by using the site-directed mutagenesis method, and the obtained TetR mutant was used to develop a fluorescence polarization assay for the determination of the 10 drugs in milk samples. Results showed that the binding affinity and the detection sensitivity of the mutant-TCs were all improved. The receptor mutation method reported here could help other researchers to evolve other receptors or recognition reagents, and the developed method could be used as a practical tool for routine screening of the residues of TCs in food samples.

## Figures and Tables

**Table 1:** Molecules, docking results, absolute affinity constants (KA) and determination parameters for the 10 TCs. The circled positions were the binding sites of the hydrophobic bond. The atoms highlighted with blue were the binding sites of the hydrogen bond.

Drug	Natural TetR					TetR Mutant					
	Binding Sites	Contact Amino Acid	Binding Energy (kcal/mol)	KA	IC_50_ (ng/mL)	Binding Sites	Contact Amino Acid	Binding Energy (kcal/mol)	KA	IC_50_ (ng/mL)	LOD (ng/mL)
MC	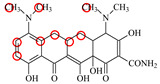	PRO105 PHE86 LEU113 LEU131	8.16	23.8	43.4	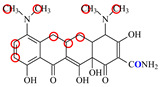	PRO105 PHE86 LEU113 LEU131 SER135	9.26	32.3	23.8	0.5
OTC	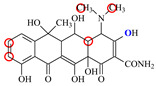	PRO105 PHE86 LEU113	8.31	21.2	59.8	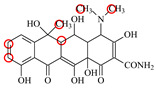	PHE86 LEU113 LEU131 SER135TRP139	7.16	29.3	36.0	0.8
TC	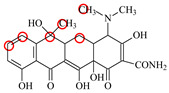	PRO105 PHE86 LEU113 LEU131	8.16	17.7	64.9	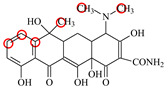	PRO105 PHE86 LEU113 LEU131	8.16	23.9	38.0	1.3
DC	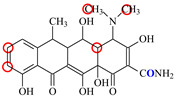	PRO105 PHE86 LEU113	7.91	24.5	36.6	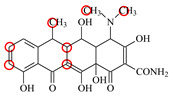	PRO105 PHE86 LEU113 LEU131 TRP139	7.4	30.5	30.3	1.0
DMC	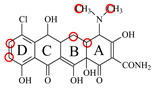	PRO105 PHE86	8.56	25.1	31	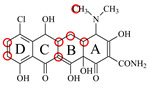	PRO105 PHE86 LEU113 SER135 TRP139	8.57	33.3	15.5	0.4
CTC	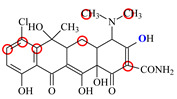	PRO105 PHE86 LEU131	8.22	18.6	80.1	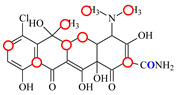	PRO105 PHE86 LEU113 LEU131 SER135	8.57	26.7	55.2	1.4
MTC	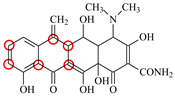	PRO105 LEU113 LEU131	7.58	22.9	50.6	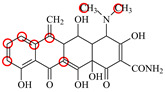	PRO105 LEU113 LEU131 TRP139	7.13	31.6	48.7	1.5
SC	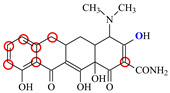	PRO105 PHE86 LEU113 LEU131	8.48	23.8	43.8	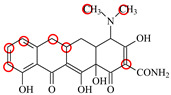	PRO105 PHE86 LEU113 LEU131 TRP139	9.11	31.1	29.0	0.7
TIC	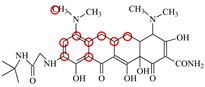	PRO105 LEU113 LEU131	7.77	25.1	30.8	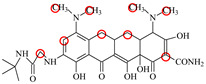	PRO105 PHE86 LEU113 LEU131	8.33	33.1	16.0	0.4
LMC	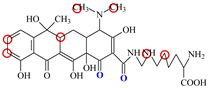	PRO105 PHE86 LEU113 LEU131	8.06	21.9	55.6	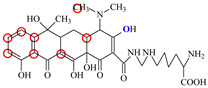	PRO105 PHE86 LEU113 LEU131 SER135 TRP139	8.47	29.8	45.7	1.0

**Table 2:** Recoveries of the 10 TCs from standard fortified blank milk samples (*n* = 6; intra-assay, mean recovery of six repetitions in one day; inter-assay, mean recovery of duplicate analysis on six successive days; coefficient of variation, standard deviation/mean value × 100%).

Drug	Added (ng/g)	Intra-Assay		Inter-Assay	
		Recovery (%)	Coefficient of Variation (%)	Recovery (%)	Coefficient of Variation (%)
MC	10	85.2	7.5	73.5	6.2
	100	93.3	8.5	86.2	8.5
TC	10	88.1	7.3	81.2	11.6
	100	87.2	7.1	93.5	9.2
OTC	10	91.3	9.5	75.7	10.6
	100	86.5	5.1	79.8	13.4
CTC	10	78.6	11.2	81.2	9.3
	100	81.2	9.4	87.4	8.7
TIC	10	85.3	8.7	76.8	16.3
	100	84.2	8.0	84.3	7.6
DC	10	90.7	6.9	71.5	6.6
	100	95.1	7.1	92.4	7.2
MTC	10	79.2	8.5	83.1	8.5
	100	78.5	9.4	91.2	9.4
LMC	10	82.4	9.3	90.2	8.6
	100	82.5	8.5	95.4	8.0
DMC	10	77.4	8.7	79.4	6.2
	100	86.8	7.9	84.4	10.5
SC	10	83.4	9.4	78.2	12.6
	100	90.5	10.3	91.2	14.3

TC, tetracycline; CTC, chlortetracycline; MC, minocycline; DC, doxycycline; OTC, oxytetracycline; DMC, demeclocycline; TIC, tigecycline; MTC, methacycline; LMC, lymecycline; SC, sancycline.

**Table 3:** Comparisons with the previous TetR-based methods for TCs.

Recognition Reagent	Method	Analyte	Limit of Detection (ng/mL)	Suitable for Screening?	Assay Time (from Add Sample)	Ref.
recombinant TetR	Chemiluminescence ELISA	5 TCs	0.005–0.016	Yes	40 min	29
TetR mutant	fluoroimmunoassay	7 TCs	0.3–0.58	Yes	30 min	30
recombinant TetR	ELISA	8 TCs	0.1–0.72	Yes	>2 h	31
recombinant TetR	dipstick	6 TCs	10	Yes	35 min	32
recombinant TetR	electrochemical chip	TC	6.33	No	15 min	33
recombinant TetR	flow-based chemiluminescence	TC	0.1	No	30 min	34
natural TetR	chemiluminescence ELISA	10 TCs	0.002–0.009	Yes	40 min	36
TetR mutant	FPIA	10 TCs	0.4–1.5	Yes	2 min	This study

## Data Availability

Data is contained within the article or Supplementary Material.

## Supplementary Materials

The following supporting information can be downloaded at: https://www.mdpi.com/article/10.3390/foods11233850/s1. Table S1: The peptide fragments of the natural TetR; Table S2: Affinity parameters of the natural TetR and the mutant for TCs; Figure S1: The mass spectrometry results of the nine peptides; Figure S2: Amino acid sequences of the natural TetR and the TetR of other bacteria; Figure S3: Docking complexes with other TCs; Figure S4: Results for optimization of the assay parameters.
