# Supplementary material for: Identification and Evolution of a Natural Tetr Protein Based on Molecular Docking and Development of a Fluorescence Polari-Zation Assay for Multi-Detection of 10 Tetracyclines in Milk
Source: Foods. 2022 Nov 28;11(23):3850. doi: 10.3390/foods11233850 (PMC9735679; doi:10.3390/foods11233850)

Supplementary Materials for

Identification and evolution of a natural TetR protein based on molecular docking and development of a fluorescence polarization assay for multi-detection of 10 tetracyclines in milk

Wan Qiu Xia, Jing Liu, Jian Ping Wang

College of Veterinary Medicine, Hebei Agricultural University, Baoding Hebei 071000, China

Table S1. The nine unique peptide fragments of the natural TetR protein.

No.	Amino Acid Sequence	Length	Mass	N-Term Cleavage Window	C-Term Cleavage Window
1	ALLDALAIEMLDR	13	1442.78	YWHVKNKRALLDALAI	LAIEMLDRHHTHFCPL
2	CALLSHR	7	855.4385	RNNAKSFRCALLSHRD	RCALLSHRDGAKVHLG
3	CALLSHRDGAK	11	1226.619	RNNAKSFRCALLSHRD	LSHRDGAKVHLGTRPT
4	EERETPTTDSMPPLLR	16	1870.91	DQEHQVAKEERETPTT	DSMPPLLRQAIELFDH
5	ETPTTDSMPPLLR	13	1456.723	HQVAKEERETPTTDSM	DSMPPLLRQAIELFDH
6	LGVEQPTLYWHVK	13	1568.835	TTRKLAQKLGVEQPTL	PTLYWHVKNKRALLDA
7	LGVEQPTLYWHVKNKR	16	1967.074	TTRKLAQKLGVEQPTL	YWHVKNKRALLDALAI
8	SFRCALLSHR	10	1245.64	DFLRNNAKSFRCALLS	RCALLSHRDGAKVHLG
9	SFRCALLSHRDGAK	14	1616.821	DFLRNNAKSFRCALLS	LSHRDGAKVHLGTRPT

Table S2. Affinity parameters of the natural TetR and the mutant for the 10 TCs.

No.	Receptor	Ligand	Ka (1/Ms)	Kd (1/s)	KD (M)	KA (Log2(KD))
1	TetR	Tetracycline	2.03E+02	1.56E-03	7.69E-06	16.988
2	TetR	Chlortetracycline	2.97E+03	1.48E-02	4.99E-06	17.612
3	TetR	Minocycline	4.54E+03	5.07E-04	1.12E-07	23.093
4	TetR	Tigecycline	1.01E+03	1.04E-05	1.04E-08	26.525
5	TetR	Doxycycline	5.34E+04	1.13E-03	2.11E-08	25.496
6	TetR	Demecycline	2.77E+03	2.06E-04	7.44E-08	23.680
7	TetR	Metacycline	1.44E+03	1.60E-04	1.11E-07	23.101
8	TetR	Lymecycline	3.17E+03	1.16E-03	3.66E-07	21.380
9	TetR	Oxytetracycline	2.06E+03	1.99E-03	9.65E-07	19.983
10	TetR	Sancycline	2.14E+03	1.27E-04	5.95E-08	24.003
11	TetR+Mg^{2+}	Tetracycline	2.78E+02	1.33E-03	4.79E-06	17.672
12	TetR+Mg^{2+}	Chlortetracycline	4.43E+03	1.13E-02	2.56E-06	18.578
13	TetR+Mg^{2+}	Minocycline	7.27E+03	5.11E-04	7.03E-08	23.763
14	TetR+Mg^{2+}	Tigecycline	6.33E+02	1.78E-05	2.82E-08	25.079
15	TetR+Mg^{2+}	Doxycycline	4.39E+04	1.80E-03	4.11E-08	24.538
16	TetR+Mg^{2+}	Demecycline	4.61E+03	1.27E-04	2.76E-08	25.110
17	TetR+Mg^{2+}	Metacycline	1.33E+03	1.62E-04	1.22E-07	22.961
18	TetR+Mg^{2+}	Lymecycline	4.59E+03	1.21E-03	2.63E-07	21.856
19	TetR+Mg^{2+}	Oxytetracycline	3.52E+03	1.44E-03	4.09E-07	21.221
20	TetR+Mg^{2+}	Sancycline	2.31E+03	1.62E-04	7.02E-08	23.764
21	TetR mutant	Tetracycline	2.03E+02	1.56E-04	7.68E-07	20.312
22	TetR mutant	Chlortetracycline	5.05E+04	8.22E-04	1.63E-08	25.871
23	TetR mutant	Minocycline	1.36E+04	4.23E-05	3.10E-09	28.265
24	TetR mutant	Tigecycline	9.09E+03	9.45E-07	1.04E-10	33.163
25	TetR mutant	Doxycycline	2.67E+05	6.65E-05	2.49E-10	31.903
26	TetR mutant	Demecycline	5.54E+03	1.72E-05	3.10E-09	28.265
27	TetR mutant	Metacycline	1.44E+03	5.71E-06	3.97E-09	27.908
28	TetR mutant	Lymecycline	2.85E+04	7.25E-05	2.54E-09	28.553
29	TetR mutant	Oxytetracycline	4.53E+04	7.96E-05	1.76E-09	29.082
30	TetR mutant	Sancycline	1.07E+04	6.35E-06	5.93E-10	30.651
31	TetR mutant+Mg^{2+}	Tetracycline	1.95E+03	1.21E-04	6.21E-08	23.941
32	TetR mutant+Mg^{2+}	Chlortetracycline	4.43E+04	4.04E-04	9.11E-09	26.710
33	TetR mutant+Mg^{2+}	Minocycline	1.67E+05	3.19E-05	1.91E-10	32.286
34	TetR mutant+Mg^{2+}	Tigecycline	1.71E+04	1.78E-06	1.04E-10	33.163
35	TetR mutant+Mg^{2+}	Doxycycline	1.76E+05	1.13E-04	6.41E-10	30.539
36	TetR mutant+Mg^{2+}	Demecycline	5.07E+04	4.70E-06	9.28E-11	33.327
37	TetR mutant+Mg^{2+}	Metacycline	1.86E+04	5.59E-06	3.00E-10	31.634
38	TetR mutant+Mg^{2+}	Lymecycline	1.01E+05	1.10E-04	1.09E-09	29.773
39	TetR mutant+Mg^{2+}	Oxytetracycline	8.10E+04	1.20E-04	1.48E-09	29.332
40	TetR mutant+Mg^{2+}	Sancycline	1.39E+04	5.79E-06	4.17E-10	31.159

Ka, association constant; Kd, dissociation constant; KD = Kd/Ka, equilibrium dissociation constant; KA = Abs (Log2(KD), absolute affinity constant.

Figure S1. The mass spectrometry results of the nine unique peptides (the number sequence was consistent with that of in Table S1).

No. 1

Raw File	Scan	Method	Score	m/z
R2176-TETR	9803	FTMS; HCD	65.5	730.39

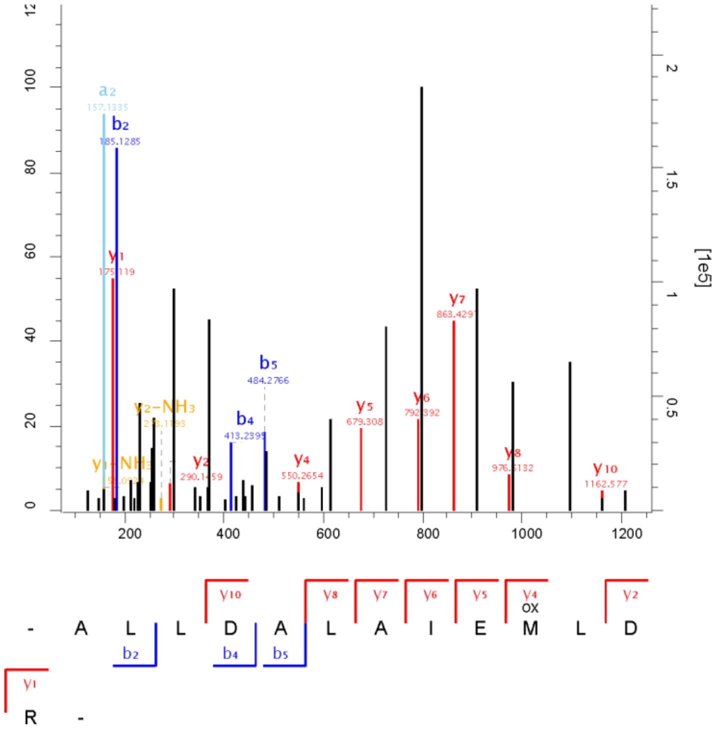

No. 2

Raw File	Scan	Method	Score	m/z
R2176-TETR	3025	FTMS; HCD	160.69	428.73

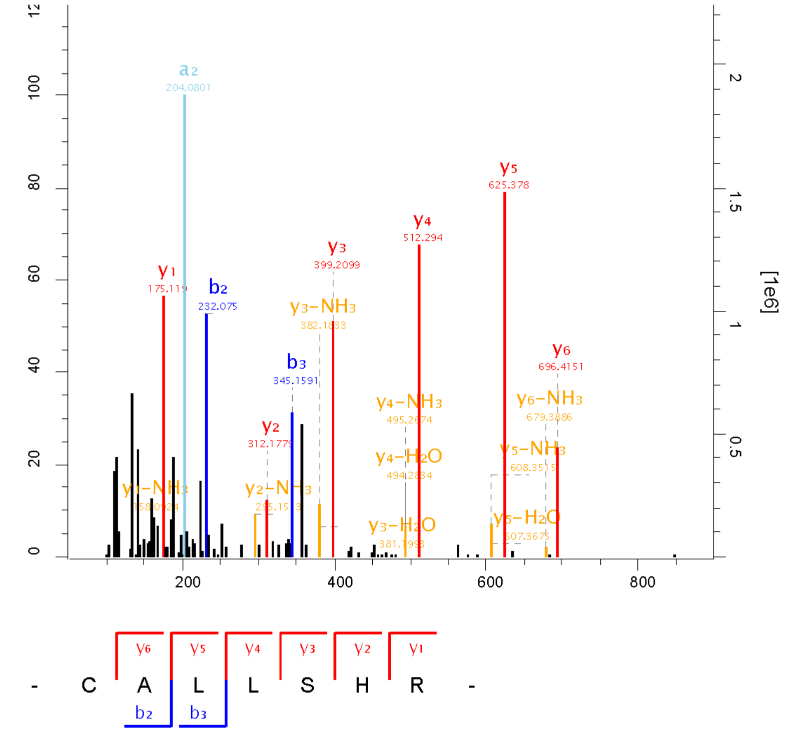

No. 3

Raw File	Scan	Method	Score	m/z
R2176-TETR	2591	FTMS; HCD	82.17	409.88

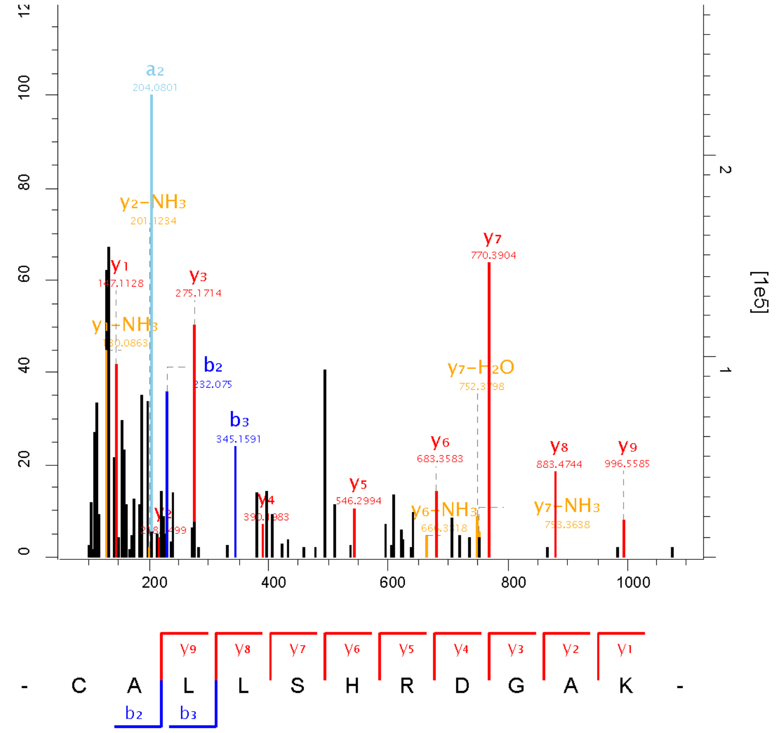

No. 4

Raw File	Scan	Method	Score	m/z
R2176-TETR	5183	FTMS; HCD	60.55	630.31

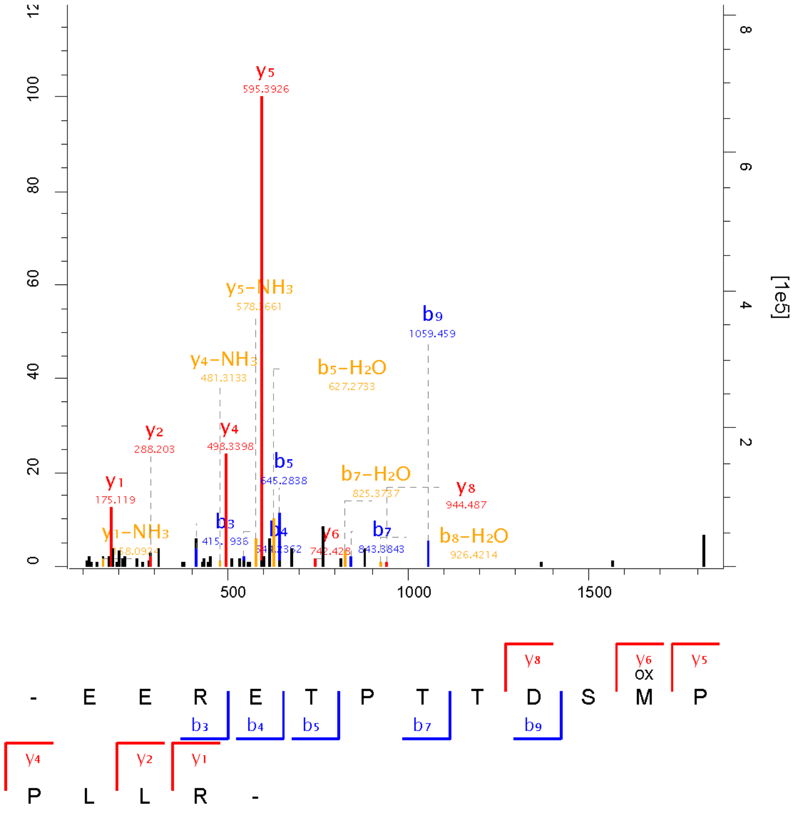

No. 5

Raw File	Scan	Method	Score	m/z
R2176-TETR	5813	FTMS; HCD	114.51	737.37

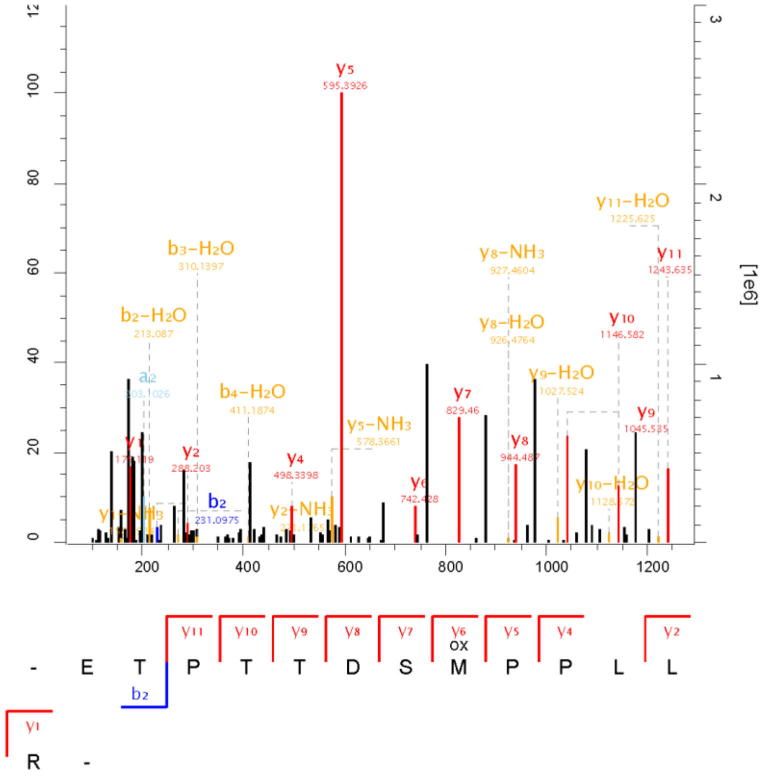

No. 6

Raw File	Scan	Method	Score	m/z
R2176-TETR	7563	FTMS; HCD	52.79	785.42

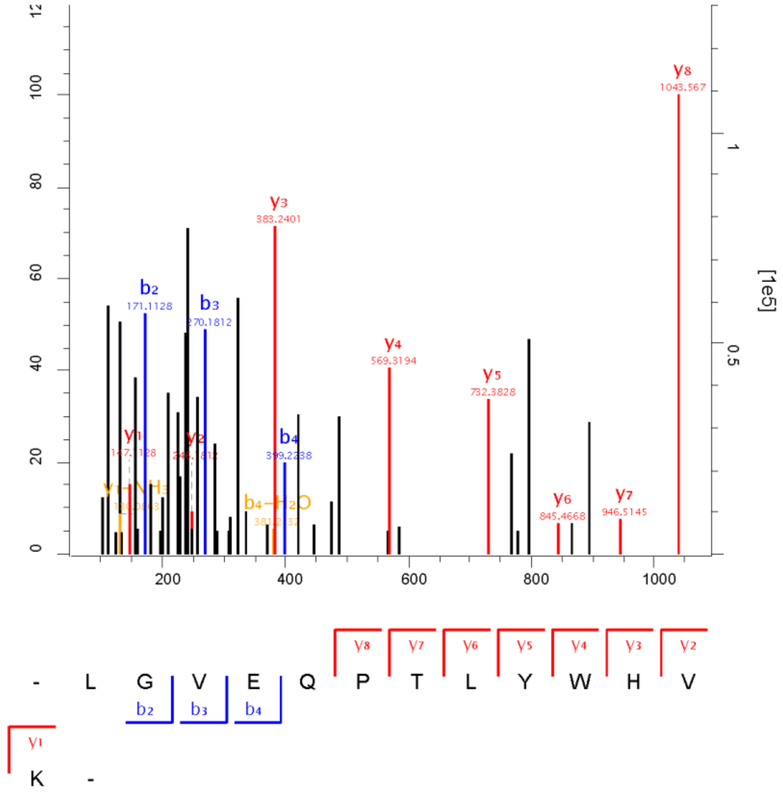

No. 7

Raw File	Scan	Method	Score	m/z
R2176-TETR	6035	FTMS; HCD	30.13	656.7

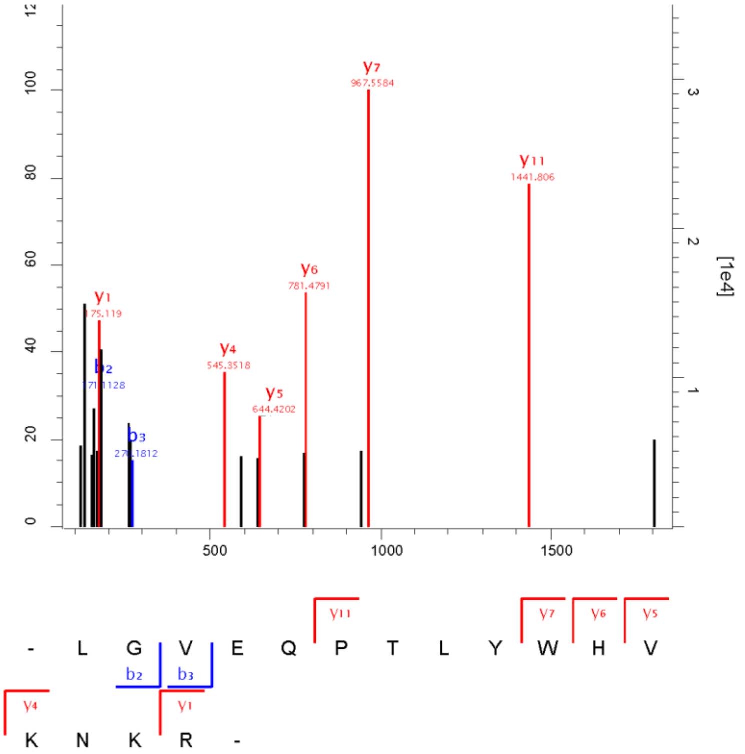

No. 8

Raw File	Scan	Method	Score	m/z
R2176-TETR	4911	FTMS; HCD	71.22	416.22

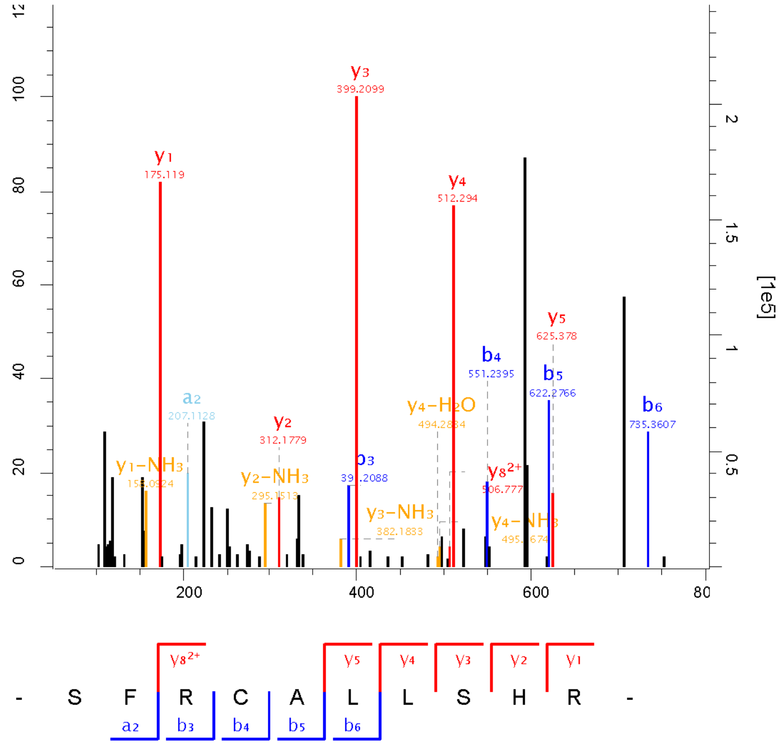

No. 9

Raw File	Scan	Method	Score	m/z
R2176-TETR	6475	FTMS; HCD	0	539.95

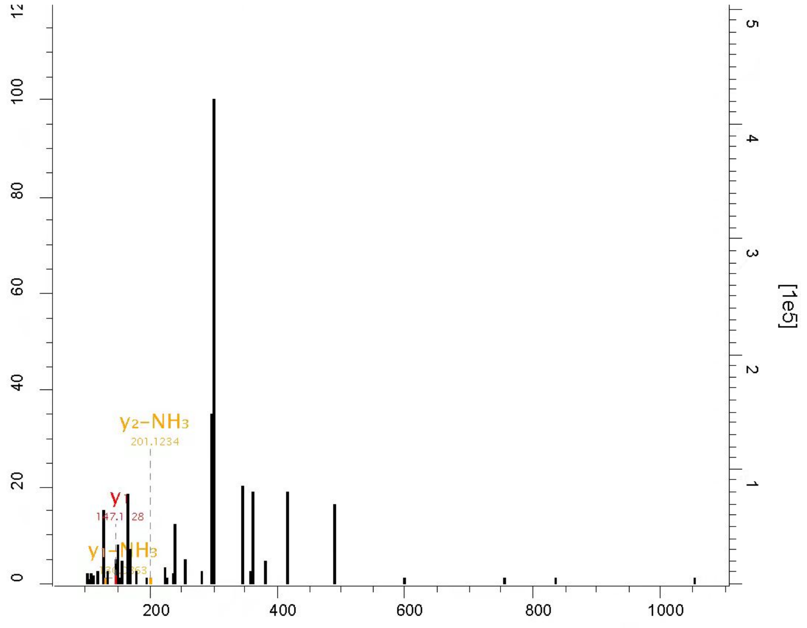

- S F R C A L L S H R D G

y1

A K -

Figure S2. Amino acid sequences of the natural TetR and the TetR from *Escherichia coli*. (NCBI ID: WP_000088605.1), *Enterobacter hormaechei* (NCBI ID: WP_058686930.1), *Morganella morganii* (NCBI ID: WP_052927103.1), *Salmonella* (NCBI ID: WP_050959760.1), and *Acinetobacter baumannii* (NCBI ID: WP_185936927.1). The highlighted amino acids are the 100% conserved residues.

```
our natural TetR.seq          MSRLDKSKVINSALELLNEVGIEGLTTRKLAQKLGVEQPTLYWHVKNKRA 50
Escherichia coli.seq          MSRLDKSKVINSALELLNEVGIEGLTTRKLAQKLGVEQPTLYWHVKNKRA 50
Enterobacter hormaechei.seq   MNKLQREAVIRTALELLNDVGMEGLTTRRLAERLGVQQPALYWHFKNKRA 50
Morganella morganii.seq       MARLNRESVIDAALELLNETGIDGLTTRKLAQKLGIEQPTLYWHVKNKRA 50
Salmonella.seq                MTKLDKGTVIAAALELLNEVGMDSLTTRKLAERLKVQQPALYWHFQNKRA 50
Acinetobacter baumannii.seq   ---------------LLNEVGVDGLTTRKLAERLGVQQPALYWHFRNKRA 35

our natural TetR.seq          LLDALAIEMLDRHHTHFCPLEGESWQDFLRNNAKSFRCALLSHRDGAKVH 100
Escherichia coli.seq          LLDALAIEMLDRHHTHFCPLEGESWQDFLRNNAKSFRCALLSHRDGAKVH 100
Enterobacter hormaechei.seq   LLDALAEAMLTINHTHSTPRDDDDWRSFLKGNACSFRRALLAYRDGARIH 100
Morganella morganii.seq       LLDALAVEILARHHDYSLPAAGESWQSFLRNNAMSFRRALLYYRDGAKVH 100
Salmonella.seq                LLDALAEAMLAERHTRSLPEENEDWRVFLKENALSFRTALLSYRDGARIH 100
Acinetobacter baumannii.seq   LLDALAEAMLAENHTHSVPRADDDWRSFLIGNARSFRQALLAYRDGARIH 85

our natural TetR.seq          LGTRPTEKQYETLENQLAFLCQQGFSLENALYALSAVGHFTLGCVLEDQE 150
Escherichia coli.seq          LGTRPTEKQYETLENQLAFLCQQGFSLENALYALSAVGHFTLGCVLEDQE 150
Enterobacter hormaechei.seq   AGTRPAAPQMEKADAQLRFLCDAGFSAGDATYALMAISYFTVGAVLEQQA 150
Morganella morganii.seq       LGTRPDEKQYDTVETQLRFMTDNGFSLRDGLYAISAVSHFTLGAVLEQQE 150
Salmonella.seq                AGTRPTEPIFCSAETQIRFLCAEGFCPKRAVWALRAVSHYVVGSVLEQQA 150
Acinetobacter baumannii.seq   AGTRPGAPQMETADEQLRFLCEAGFSAGDAVNALMTISYFTVGAVLEEQA 135

our natural TetR.seq          HQVAKEERE----TPTTDSMPPLLRQAIELFDHQGAEPAFLFGLELIICG 196
Escherichia coli.seq          HQVAKEERE----TPTTDSMPPLLRQAIELFDHQGAEPAFLFGLELIICG 196
Enterobacter hormaechei.seq   SEADAEERGEDQLTTSASTMPARLQSAMKIVYEGGPDAAFERGLALIIGG 200
Morganella morganii.seq       HSAALADRS----ATPDENLPPLLREALHIMDSDDGEQAFLHGLESLILG 196
Salmonella.seq                S--DADERVPDRPDVSEQAPSSFLHDLFHELETDGMDAAFNFGLDSLIAG 198
Acinetobacter baumannii.seq   GDSDAGERGG---TVEQAPLSPLLRAAIDAFDEAGPDAAFEQ         174

our natural TetR.seq          LEKQLKCESGS                                        207
Escherichia coli.seq          LEKQLKCESGS                                        207
Enterobacter hormaechei.seq   LEKMRLTTNDIEVLKNVDE                                219
Morganella morganii.seq       FEVQLTHSGQSPQ                                      209
Salmonella.seq                FERLRSSTTD                                         208
Acinetobacter baumannii.seq                                                      174
```

Figure S3. Docking complexes of the natural TetR (A-I) and the mutant (A’-I’) with other 9 TCs.

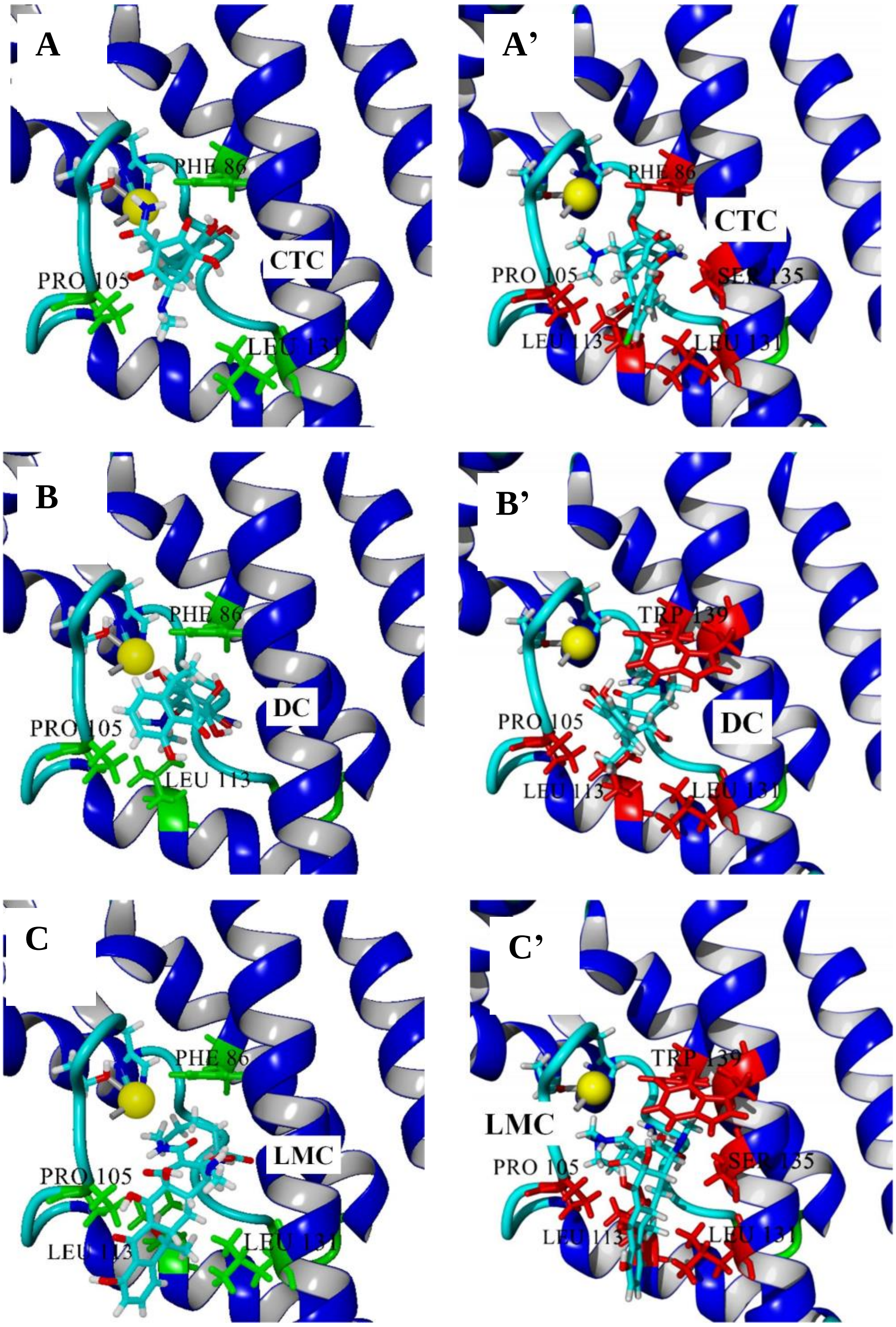

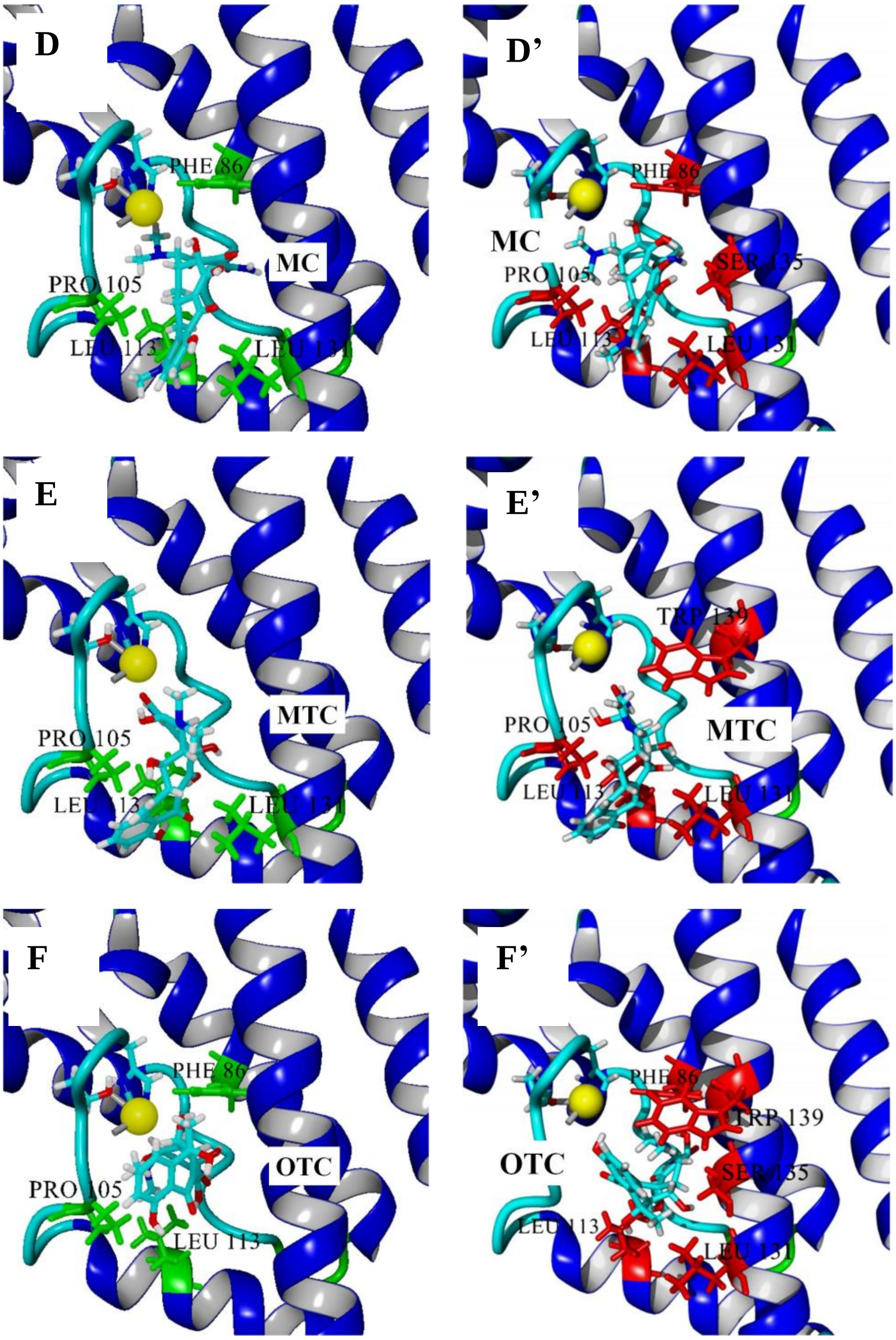
D
PHE 86
MC
PRO 105
LEU 113
LEU 131
D'
PHE 86
MC
PRO 105
SER 135
LEU 113
LEU 131
E
MTC
PRO 105
LEU 113
LEU 131
E'
TRP 139
PRO 105
MTC
LEU 113
LEU 131
F
PHE 86
OTC
PRO 105
LEU 113
F'
PHE 86
TRP 139
OTC
SER 135
LEU 113
LEU 131

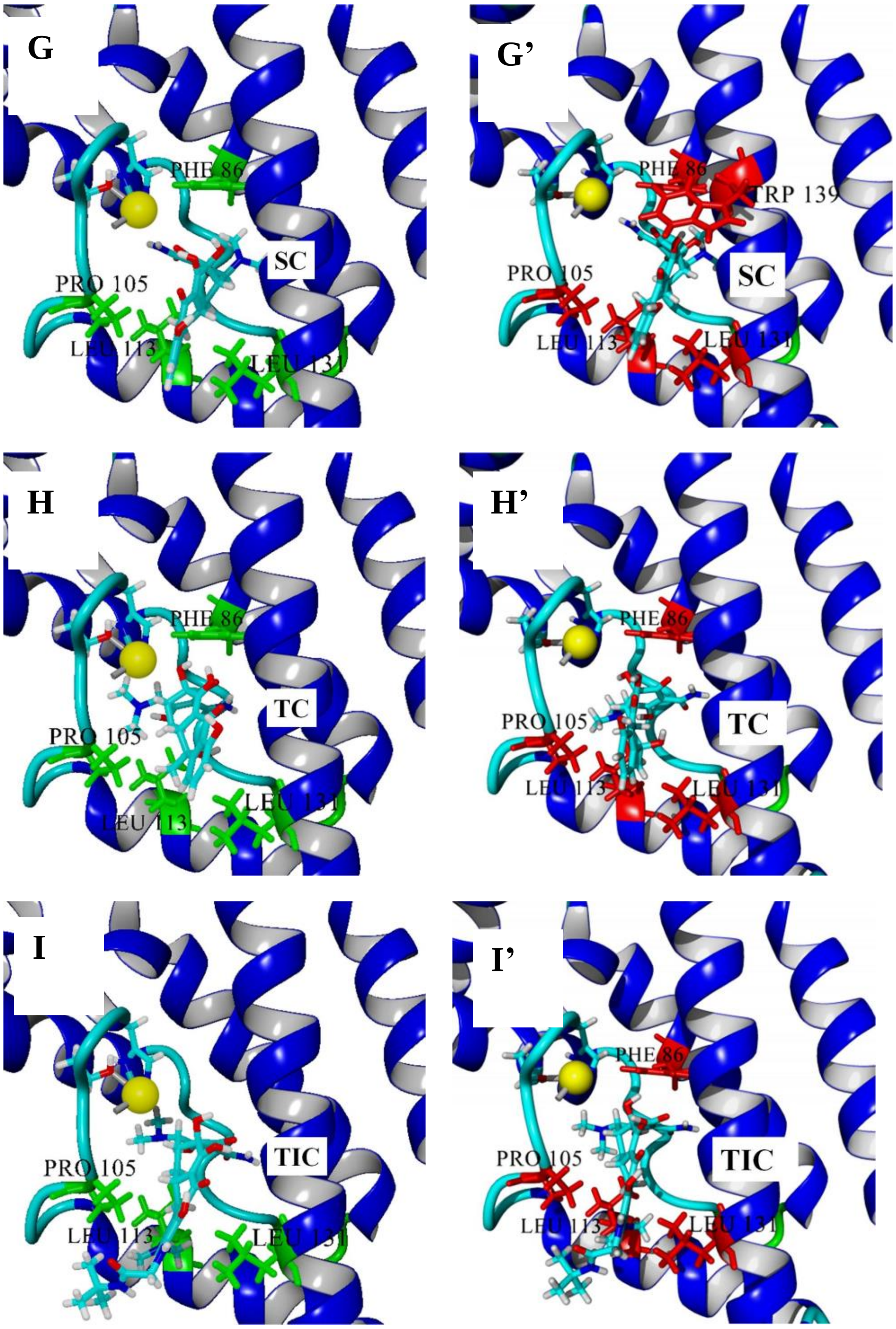
G
PHE 86
SC
PRO 105
LEU 113
LEU 131
G'
PHE 86
TRP 139
SC
PRO 105
LEU 113
LEU 131
H
PHE 86
TC
PRO 105
LEU 113
LEU 131
H'
PHE 86
TC
PRO 105
LEU 113
LEU 131
I
TIC
PRO 105
LEU 113
LEU 131
I'
PHE 86
TIC
PRO 105
LEU 113
LEU 131

Figure S4. Results for optimization of (A) FA-MC/mutant concentrations ($MgCl_2$ 5 mM, incubation 5 min), (B) $MgCl_2$ concentration (FA-MC 1:500, mutant 1:2000, incubation 5 min), and (C) incubation time (FA-MC 1:500, mutant 1:2000, $MgCl_2$ 8 mM) by using 100 ng/mL of DMC (n=5).

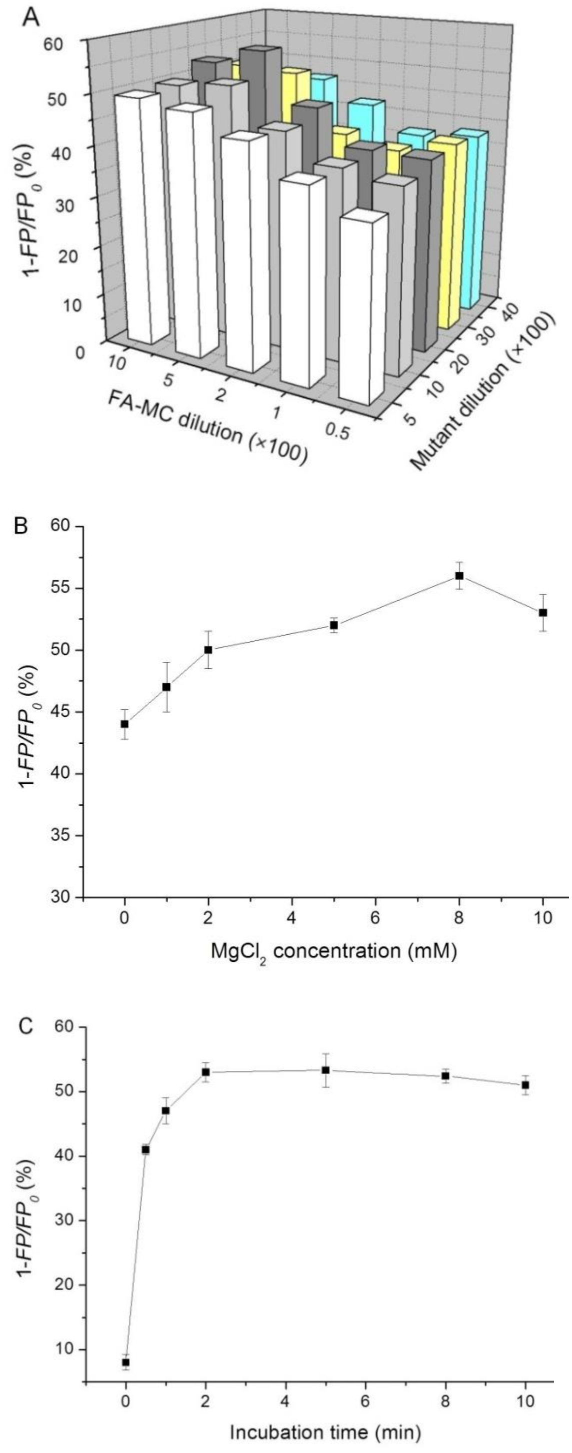

Supplement: Supplementary file 1 [file foods-11-03850-s001.zip › foods-1954752-supplementary.pdf]
